# Comparing Multiplex PCR and Rapid Urease Test in the Detection of *H. pylori* in Patients on Proton Pump Inhibitors

**DOI:** 10.1155/2012/898276

**Published:** 2012-12-23

**Authors:** Thomas Chen, Xiangwen Meng, H. Zhang, Rebecca W. Tsang, Tat-Kin Tsang

**Affiliations:** ^1^College of Medicine, University of Illinois at Chicago, 1853 West Polk Street, Chicago, IL 60612, USA; ^2^TZAM Diagnostics LLC, 1824 Wilmette Avenue, Wilmette, IL 60091, USA; ^3^Department of Medicine, Loyola University Hospital, Maywood, IL 60153, USA; ^4^Department of Gastroenterology, NorthShore University Health System, University of Chicago, Glenview, IL 60026, USA

## Abstract

*Background*. This study was conducted to assess the diagnostic value of a multiplex PCR assay to detect *H. pylori* infection and to further evaluate the negative results from the CLOtest on patients with and without PPI treatment. Methods. This study is a retrospective cohort that included 457 patients with symptoms of dyspepsia, who underwent upper endoscopy at Evanston and Glenbrook Northshore Hospital from June 2003 to October 2007. A total of 556 samples were reported with some patients having more than one test over the time period. The CLOtest was performed first on the gastric specimen and from that specimen, the DNA was isolated and the one-step multiplex PCR was performed. *Results*. By M-PCR testing*, H. pylori* was detected in 143 (52.2%) of 274 cases in the control group and 130 (46.1%) of 282 cases in patients on PPI treatment (*P* = 0.1746). The CLOtest detected the presence of *H. pylori* in 4 (1.4%) of 282 cases from the same group receiving PPI treatment and 29 (10.6%) of 274 cases from the group not taking a PPI (*P* ≤ 0.0001). *Conclusion*. Our PCR is sensitive enough to detect the presence of *H. pylori* despite being on PPI treatment.

## 1. Purpose

*Helicobacter pylori* (*H. pylori*) is a spiral-shaped bacterium that is found primarily in the stomach [1]. The bacteria has a significant pathogenic role in gastritis, gastric cancer, gastric mucosa-associated lymphoid tissue lymphoma, and peptic ulcer disease [2]. The World Health Organization classifies *H. pylori* as a class I carcinogen [3]; this is a major concern because approximately half of the world's population are infected with *H. pylori* [4].

Currently, there are numerous tests available to identify *H. pylori*, but there is no gold standard. Rapid urease test (RUT) is widely used in clinical practice to detect the urease enzyme of *H. pylori* in gastric mucosal biopsies. The urease enzyme hydrolyzes urea into carbon dioxide and ammonia allowing *H. pylori* to survive in the acidic medium [2, 5]. It is commonly believed that acid-reducing drugs, in particular proton pump inhibitors (PPIs), decrease the sensitivity and accuracy of the RUT, the urea breath test, histology, and the stool antigen test by reducing the amount of *H. pylori* [6, 7].

Proton pump inhibitors decrease the activity of *H. pylori* within the stomach and shift their distribution proximally. It is proposed that PPIs inhibit the growth of *H. pylori* through a pH-dependent mechanism. Proton pump inhibitors can cause false negatives in diagnostic tests and should be stopped for at least 2–4 weeks before performing a test [8, 9]. However, this generates a problem because PPI withdrawal is strongly associated with symptom recurrence. While on a PPI, a negative RUT is insufficient to rule out an infection. The biopsy specimen may contain low bacterial density of viable cells, giving a negative result. This becomes an issue as many Americans are taking these medications. In 2009, PPIs ranked third in US sales and sixth in the total numbers of prescriptions dispensed [10]. In several studies, the authors concluded that PPIs reduce the sensitivity and specificity of the antral and corpus biopsies for RUT and histological examination. The polymerase chain reaction (PCR) is more sensitive in detecting *H. pylori*. Yakoob et al. showed that the PCR is more sensitive than RUT and histology in patients taking PPIs [7, 11, 12]. However, the problem of single-gene PCR still has less than ideal specificity and false positives. With the problems that acid-reducing drugs cause to many diagnostic tests of *H. pylori*, the mutation rates of DNA, and current PCR methods testing for 1 or 2 genes, we developed a unique multiplex PCR (M-PCR) that detects 5 unique genes, improving the specificity. 

In a previous study we conducted, our unique M-PCR accurately identifies *H. pylori* compared to RUT and immunohistochemical analyses; in addition to identifying significant number of *H. pylori* infections that would not be detected by the former methods [13–15]. The aim of this study is to determine the effect of PPIs on the results of the RUT and M-PCR. We hypothesize that M-PCR will not be affected by the physiological changes from PPIs due to the sensitivity of M-PCR technology and stability of DNA.

## 2. Methods

### 2.1. Patients

This study is a retrospective cohort that included 457 patients with symptoms of dyspepsia, who underwent upper endoscopy at Evanston and Glenbrook Northwestern Hospital from June 2003 to October 2007. Biopsies were taken at the gastric antrum and body. The study was divided into two groups based on a comprehensive chart review: the first group was on a PPI and the control group was not on a PPI for at least four weeks. Those taking H_2_-receptor antagonists and antibiotics within the past 4 weeks before the endoscopy were excluded from both groups. Informed consent was obtained from each patient, and the study was reviewed and approved by the Evanston Northwestern Health Care Institutional Review Board.

### 2.2. Rapid Urease Test (CLOtest)

The CLOtest rapid urease test (Kimberly-Clark, Roswell, GA, USA) was performed first on all the gastric specimens according to the manufacturer's instructions. A definite magenta color was required to read the test as positive. The results were interpreted after 20 minutes and then 24 hours later.

### 2.3. Multiplex PCR

**Figure 1:**
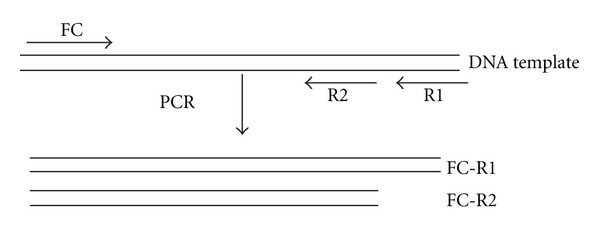
Diagram of primers designed for each locus. FC, the forward primer, is the common primer; R1 and R2 are the pair of reverse primers. The amplicons FCR1 and FC-R2 are amplified from each locus.

**Figure 2:**
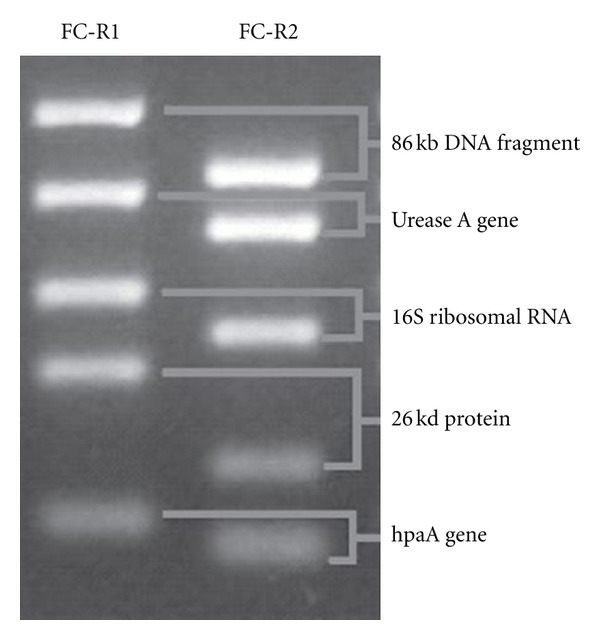
Five pairs of DNA bands amplified from the 5 targeted loci specific for *Helicobacter pylori*.

After the CLOtest was read, the same specimen was sent to the laboratory to isolate the DNA. Then the one-step M-PCR was performed. The researcher evaluating the M-PCR electrophoresis gel was blinded to the CLOtest results. The M-PCR targeted the following loci: 0.86-kb DNA fragment, urease A gene, 16S ribosomal RNA, 26-kDa protein antigen, and hpaA gene. For each locus, one forward primer, the common primer (FC), and two reverse primers (R1 and R2) were selected. The R2 primer is located inside the amplifying region of R1. The R1 and R2 primers were mixed with five FC primers, respectively, and set in two separate amplification systems of FC-R1 and FC-R2 primers (Figure 1). A total of 10 DNA fragments could be amplified, in 2 tubes, each containing 5 amplicons internal to the other. For the M-PCR, we define a positive case for *H. pylori* if 5 of the 10 fragments or two sets of DNA fragments from the same locus were amplified because of the high diversity of DNA sequences of the bacteria (Figure 2) [13–16]. In each M-PCR run, positive (strain J99) and negative (water blank) control samples are assayed to ensure that there is a reference and no contamination. Each M-PCR performed contains a negative control that contains all reaction components except gastric tissue to assess for contamination. Also, three physically separate work places were set up for template preparation, PCR reactions, and post-PCR analysis to avoid contamination. Special aerosol-resistant pipette tips and routine UV and alcohol cleaning were used. 

### 2.4. Statistical Analysis

The statistical package used was Graph InStat Version 3.10. Statistical analysis was performed by using the Fisher Exact test, 2-tailed. *P* values of less than 0.05 were considered significant.

## 3. Results

A total of 556 samples were reported with some patients having more than one test over the time span in which data was collected. A postclinical record review indicated that there were 282 (50.7%) cases where people were taking a PPI before endoscopy for at least four weeks.

There was no difference between the two groups tested by M-PCR. By M-PCR testing, *H. pylori* was detected in 143 (52.2%) of 274 cases in the control group and 130 (46.1%) of 282 cases in patients on PPI treatment (*P* = 0.1746). The CLOtest detected the presence of *H. pylori* in 4 (1.4%) of 282 cases from the same group receiving PPI treatment and 29 (10.6%) of 274 cases from the group not taking a PPI (*P* ≤ 0.0001). The M-PCR identified *H. pylori* in 33 (97.1%) of the 34 cases from the CLOtest (Table 1).

In both the PPI and no PPI groups, there was a significant difference in detection rates between the CLOtest and M-PCR (*P* ≤ 0.0001). Additional 241 (46.1%) of 523 cases were detected by M-PCR that were CLOtest negative. Specifically in the PPI group, 127 additional cases out of 278 (45.7%) were detected and 114 out of 245 (46.5%) in the control group were detected.

## 4. Conclusion

*H. pylori* is known to be a major human pathogen. Because of the diverse effects of *H. pylori*, an accurate detection method is needed. Currently, there is no one method that is sufficiently sensitive and specific to be considered “gold standard,” so we could not use a standard to compare; but used what is commonly used in practice with proven clinical significance. We developed a unique multiplex PCR assay to detect *H. pylori* in endoscopic biopsy specimens.

This study has demonstrated that PPIs affect the *H. pylori* detection rate by CLOtest method, but not the M-PCR. This is an important factor to consider when choosing a diagnostic test to detect *H. pylori*. Also, the results showed additional 46.1% positive cases by retesting the negative results by the CLOtest. Therefore, *H. pylori* testing by current methods should be carefully reviewed, especially the patients who have recently been taking PPIs to ensure that the result is not a false-negative. The CLOtest is highly specific but requires a high density of bacteria for detection. The M-PCR is sensitive enough to detect the presence of *H. pylori* despite an individual being on PPI treatment. 

The high detection rate of the M-PCR in our study can be attributed to our study patient population. We only tested patients that were symptomatic, which are more likely to have an infection; as a result the numbers would be higher in these types of patients. Another source for the difference in detection rates and low detection rates in the RUT between the groups is from the formation of the coccoid forms of *H. pylori*. It can exist in three stages: spiral, viable coccoid, and degenerative unviable coccoid form. The coccoid forms can be induced by various conditions, such as PPI and certain antibiotics [17]. Studies have shown that the protein content and genetic material remain unchanged during the conversion from spiral to coccoid forms. The urease activities of the coccoid cells are lower than the spiral form [18]. Identification of the coccoid forms by RUT is difficult; however, PCR methods are used to detect the genetic material since DNA stays intact. A study by Can et al. showed the reliability of the ureA gene region in the coccoid form, which was induced by different factors since no mutations were detected [19]. We have the assumption shown by previous studies that our M-PCR is able to detect the DNA in the coccoid form. The coccoid form may be less virulent and less likely to colonize and induce inflammation. However, it may play a role in infection and is suspected to be partly responsible for relapse of infection after antimicrobial treatment. When the conditions become suitable, the coccoid form can revert back to the spiral form and may regain infectivity [18].

We did not exclude other medications other than the ones mentioned. Any drug that increases pH or an antibiotic can affect the growth of *H. pylori* thus possibly affecting the results. One patient tested positive by the CLOtest, while negative by the M-PCR. There could be several reasons for this discrepancy; it could be a false positive where the CLOtest is 97% specific or where a different urease producing enzyme bacteria or the M-PCR could not detect the bacteria due to a gene mutation. In our previous study, all the positive patients by immunohistochemical analysis and the CLOtest were also positive through the M-PCR method in gastric specimens [13].

Our positive M-PCR results of 46.1% and 52.2%, PPI and control groups, respectively, are similar to our previous study that detected 52% of the cases with *H. pylori* and an additional 40% from the negative results. We conducted a blinded study that correlated the detection rates of the M-PCR to inflammation scores, immunohistochemical findings, and CLOtest results. The M-PCR and CLOtest results were not known by an independent pathologist who examined the histological features. The study concluded that in gastric biopsy specimens the average activity and chronic inflammatory scores were significantly greater in PCR-positive than in PCR-negative, showing the presence of *H. pylori*. In the gastric biopsy specimens, the M-PCR detected *H. pylori* in all the positive cases detected by immunohistochemical analysis and/or CLOtest [13]. 

The results of the study and findings are consistent with those of a previous study conducted by Yakoob et al. The study found no difference in the detection rates by a PCR between the group that was on a PPI and the control group, 74% versus 75% in the antrum. It also concluded that the diagnostic yield of both RUT and histology was reduced and PCR is more sensitive than both. In the PPI group biopsied from the antrum, 74% in the PCR, 18% in the RUT, and 50% in the histology were tested positive. Also, additional 68% in the PPI group and 44% in control were found to be positive by PCR. The detection rates for the PCR are higher compared to our study and this can be due to the smaller study population, ethnic background, or sicker patients. Also, the detection rate may be higher due to the fact that their study used one gene versus our studying using 5 genes. The study concluded that the use of acid reducing drugs decreased the diagnostic yield of RUT compared to the PCR and histology [11]. In most other studies involving traditional PCR, one or two genes were used to identify *H. pylori*. Our M-PCR differs from all other studies since our M-PCR uses 5 genes, and therefore, is more specific for *H. pylori*. So it may be difficult to correlate the results of our M-PCR to traditional PCR.

The use of PCR technology for detection of microorganisms, including *H. pylori*, is well documented. Potential problems with traditional PCR methods include false positives due to contamination and homological DNA sequences among various species. In our study, there was a large difference between the M-PCR and CLOtest in the non-PPI group that may represent false positives [20]. The primers used for the M-PCR were scanned across the gene bank of the National Center for Biotechnology Information and no matches were found. Also the M-PCR for *H. pylori* was tested against 19 bacterial species (*E. coli, Enterobacter aerogenes, Enterobacter cloacae, Enterococcus *species,* Viridians *Group,* P. aeruginosa, Serratia *species,* Klebsiella pneumoniae, MRSA, Lactobacillus *species,* Citrobacter *species,* Bacteroides fragilis (ATCC25285), Wolinella succinogenes (ATCC29543), Campylobacter Jejuni (ATCC33291), Helicobacter pullorum (ATCC52802), Helicobacter fennelliae (ATCC35683), Helicobacter *species* (ATCC35683), Helicobacter heilmannii (ATCC49286), and Helicobacter felis (ATCC49179*)). None of the 19 bacteria showed the standard *H. pylori* M-PCR band patterns. There is also an internal control to prevent false positives. Our M-PCR amplifies 10 DNA fragments at the same time as well as two fragments that will be produced for each of the five loci, one internal to the other. The internal control of our one-step multiple-nested PCR is used to rule out false-positives caused by homological DNA sequences among various species in the primer binding sites, making the M-PCR more specific than the traditional PCR. Also as previously stated, our M-PCR test was validated through our study that showed that our positive M-PCR results showed significantly greater average activity and chronic inflammatory scores.

The detection rates of RUT in non-PPI group were low in the study. This may have been attributed to other acid-reducing medications we did not exclude like bismuth compounds or calcium products. Although we went through a comprehensive medication review and survey, patients may not have disclosed full information. Our detection rates were lower than other studies, but other studies mentioned have yielded low rates as well [11, 12].

Overall, the M-PCR detected an additional 241 positive cases. If a patient has a negative result from a RUT, our M-PCR has proven useful in the diagnostics of *H. pylori* to further evaluate the negative result, as it is not affected by acid reducing drugs. The use of M-PCR can be recommended as an additive test to confirm the presence of *H. pylori* in patients with a negative RUT. Also, for clinicians who require their patients to be on empirical treatment or maintenance therapy, the M-PCR assay can be used so the patient does not need to be taken off a PPI. This M-PCR assay identified a significant number of *H. pylori* infections that would not be detected by RUT, finding additional 46.1% positive cases. Our M-PCR for *H. pylori* will increase detection rates, increasing opportunities for medical interventions and allowing for patients to be treated through sensitive and specific method that is not affected by PPI unlike many other diagnostic methods. We developed an available M-PCR in the United States available for physicians to utilize.

## Figures and Tables

**Table 1:** Difference between M-PCR and CLOtest.

	On PPI	Without PPI	*P* value
	n = 282		
CLOtest			
Positive	4 (1.4)	29 (10.6)	<0.0001
Negative	278 (98.6)	245 (89.4)	
M-PCR			
Positive	130 (46.1)	143 (52.2)	0.1746
Negative	152 (53.9)	131 (47.8)	
